# Incorporating parent-of-origin effects in whole-genome prediction of complex traits

**DOI:** 10.1186/s12711-016-0213-1

**Published:** 2016-04-18

**Authors:** Yaodong Hu, Guilherme J. M. Rosa, Daniel Gianola

**Affiliations:** Department of Animal Sciences, University of Wisconsin-Madison, 1675 Observatory Dr., Madison, WI 53706 USA; Department of Biostatistics and Medical Informatics, University of Wisconsin-Madison, 600 Highland Avenue, Madison, WI 53792 USA; Department of Dairy Science, University of Wisconsin-Madison, 1675 Observatory Dr., Madison, WI 53706 USA

## Abstract

**Background:**

Parent-of-origin effects are due to differential contributions of paternal and maternal lineages to offspring phenotypes. Such effects include, for example, maternal effects in several species. However, epigenetically induced parent-of-origin effects have recently attracted attention due to their potential impact on variation of complex traits. Given that prediction of genetic merit or phenotypic performance is of interest in the study of complex traits, it is relevant to consider parent-of-origin effects in such predictions. We built a whole-genome prediction model that incorporates parent-of-origin effects by considering parental allele substitution effects of single nucleotide polymorphisms and gametic relationships derived from a pedigree (the POE model). We used this model to predict body mass index in a mouse population, a trait that is presumably affected by parent-of-origin effects, and also compared the prediction performance to that of a standard additive model that ignores parent-of-origin effects (the ADD model). We also used simulated data to assess the predictive performance of the POE model under various circumstances, in which parent-of-origin effects were generated by mimicking an imprinting mechanism.

**Results:**

The POE model did not predict better than the ADD model in the real data analysis, probably due to overfitting, since the POE model had far more parameters than the ADD model. However, when applied to simulated data, the POE model outperformed the ADD model when the contribution of parent-of-origin effects to phenotypic variation increased. The superiority of the POE model over the ADD model was up to 8 % on predictive correlation and 5 % on predictive mean squared error.

**Conclusions:**

The simulation and the negative result obtained in the real data analysis indicated that, in order to gain benefit from the POE model in terms of prediction, a sizable contribution of parent-of-origin effects to variation is needed and such variation must be captured by the genetic markers fitted. Recent studies, however, suggest that most parent-of-origin effects stem from epigenetic regulation but not from a change in DNA sequence. Therefore, integrating epigenetic information with genetic markers may help to account for parent-of-origin effects in whole-genome prediction.

## Background

Parent-of-origin effects are asymmetric influences that act on phenotype of offspring, depending on the sex of the parent. Genomic imprinting, manifested as differential and/or preferential gene expression that is usually caused by differential DNA methylation [1, 2] or histone modification [3] on different parental alleles, is one of the most studied epigenetic mechanisms and an important source of parent-of-origin effects. Imprinting has an impact on several human diseases [4–8] such as the Prader–Willi (PWS) and Angelman (AS) syndromes [9, 10], as well as on complex traits in livestock [11–13]. For example, mapping studies have detected presumably imprinted quantitative trait loci (QTL) that affect economically important traits in swine [14–21], beef cattle [22–24], sheep [25], mice [26, 27], and dogs [28]. In addition, genome-wide scan studies with dense single nucleotide polymorphism (SNP) chips have also suggested that imprinted loci are associated with complex traits in various mammalian species (e.g., [29–34]).

QTL mapping studies can identify genomic regions that contribute to traits of interest and to marker assisted selection (MAS, [35, 36]). However, use of QTL mapping for breeding purposes has failed to yield clear dividends (e.g., [37, 38]). A possible explanation is that QTL mapping studies require, e.g., carefully designed crossbreeding experiments and these are seldom available in livestock. Thus, artificial selection using predicted genetic merit of selection candidates is still mainly used in animal improvement programs. Breeding values have been predicted based on resemblances between relatives using pedigree information (e.g., [39, 40]). In the genomics era, however, the availability of high-throughput genotyping techniques makes it possible to interrogate genotypes of hundreds of thousands or even millions of SNPs simultaneously, resulting in what is known as “genomic selection” or “whole-genome prediction” [41–43]. With continuously decreasing genotyping costs, genomic selection has become affordable for commercial settings in some species [44], and QTL mapping is less used in animal breeding, unless the objective is to find a major gene. Even in crops, genomic selection is gradually replacing QTL-MAS. Although some debate persists [45], genomic selection will probably be the main approach used in the foreseeable future [46].

Genomic selection (GS) and whole-genome prediction (WGP) exploit associations between phenotypes and an enormous number of SNPs under certain statistical assumptions regarding the underlying trait architecture. Often, the association between phenotype and SNPs is explored by using the SNPs as covariates in a linear regression model. Since the number of covariates (*p*) is usually much larger than the number of observations (*n*), different techniques have been used to circumvent the “curse of dimensionality” in GS/WGP studies. Commonly used methods include Bayesian regression (e.g., [41, 43, 47, 48]), G-BLUP (e.g., [49, 50]), semi-parametric methods (e.g., [51–54]) and neural networks (e.g., [55–57]), among others. All these models assume that the inheritance of the complex trait is Mendelian, i.e., paternally- and maternally-inherited alleles are functionally equivalent. Under this assumption, no phenotypic difference between genotypes $A_1A_2$ and $A_2A_1$ is expected. SNPs are assigned codes such as 0, 1 or 2 according to genotype at the locus, and the average substitution effects of all markers in the model are estimated simultaneously. Prediction is then performed by combining the estimates of these SNP effects with a genotype matrix in an independent set of individuals. However, recent studies suggest that some traits are not strictly Mendelian. For example, Mott et al. [58] found that 91 out of 97 murine traits were subject to parent-of-origin effects. In a review, Lawson et al. [13] also suggested that parent-of-origin effects may be more prevalent than previously thought. Perhaps parent-of-origin effects may enhance WGP models, if considered appropriately.

Currently used GS models may not be suitable for parent-of-origin-effects-affected traits, for which inheritance of one allele from the father may have a different effect on the phenotype than when the same allele is inherited from the mother. This suggests that two distinct substitution effects associated with the two parental origins of an allele are needed. A one-locus quantitative genetic model that takes imprinting into account has been proposed [17, 59, 60], where genotypes $A_2A_2$, $A_1A_2$, $A_2A_1$ and $A_1A_1$ are assumed to have genotypic values $-a$, $d-i$, $d+i$ and *a*, respectively, and paternal and maternal allele substitution effects are defined as $\alpha _{\mars } = a+d(q-p)+i$ and $\alpha _{\venus } = a+d(q-p)-i$. In a previous study, a genome-wide association study (GWAS)-like scan conducted with this model indicated that ignoring imprinting may underestimate additive genetic variation [61], which suggested that prediction accuracy may be higher when imprinting is considered in WGP. In a recent simulation study, Nishio and Satoh [62] suggested that the unbiasedness of variance component estimation may be enhanced when imprinting is integrated under a genomic best linear unbiased prediction (G-BLUP) framework. Here, we build a prediction model that incorporates parent-of-origin effects parametrically and assess whether or not this model improves prediction of phenotypes over the additive model currently employed in WGP using real data. In addition, we evaluate the advantages and limitations of the full model using simulated data and give a detailed discussion on its application under various conditions. Our study complements that of [62] and provides insights into prediction with parent-of-origin effects from a Bayesian perspective.

Before proceeding, some clarification is necessary. In much of the epigenetic literature, the terms “imprinting effects” and “parent-of-origin effects” have been used interchangeably. In “*i*QTL mapping” studies, for example, the detected QTL are putatively imprinted. However, the statistical model used in *i*QTL mapping does not guarantee that the detected parent-of-origin effects are necessarily due to imprinting. A counter-example was presented by Hager et al. [63], where maternal effects can mimic imprinting effects in statistical analysis. Furthermore, parent-of-origin effects were detected in birds [64, 65], although no strong evidence of imprinting in birds is available [66–68]. Thus, results obtained from the model described herein and its variants should be interpreted as parent-of-origin effects but not beyond [61]. In this study, we build WGP models to incorporate parent-of-origin effects, aiming at obtaining a higher predictive accuracy when a complex trait is subject to parent-of-origin effects. We use the term “parent-of-origin effects” throughout, but in the simulations we mimicked imprinting mechanisms to simplify the source of parent-of-origin effects. The simulated data was used for model evaluation under various conditions.

This paper is organized as follows. First, a previously proposed mixed effect model that incorporates parent-of-origin effects at the lineage level is introduced. Then, a brief introduction of a one-locus quantitative genetic model that takes genomic imprinting into account is provided. We extend this model to incorporate all available SNPs simultaneously to include parent-of-origin effects at the DNA (SNP) level, and our prediction model is constructed using both pedigree and DNA information. This model is applied to real (mouse) and simulated data and its predictive performance is compared to that of an additive model. Following a section that discusses advantages and drawbacks of this model, a discussion on the possibilities and challenges of conducting whole-genome prediction models that use epigenetic information to incorporate parent-of-origin effects is provided.

## Prediction model incorporating parent-of-origin effects

Consider the pedigree-based additive effects model (e.g., [39, 40]):

1 $$\begin{aligned} {\mathbf {y}} = {\mathbf {1}}\mu + {\mathbf {Xb}} + {\mathbf {Zu}} + {\mathbf {e}}, \end{aligned}$$

where the $n\times 1$ vector ${\mathbf {y}}$ contains phenotypic records; $\mu$ is an effect common to all individuals; ${\mathbf {b}}$ is a vector of fixed effects with associated incidence matrix ${\mathbf {X}}$; ${\mathbf {u}}$ is the $n\times 1$ vector of normally distributed infinitesimal additive effects with zero mean vector and variance–covariance matrix ${\mathbf {A}}\sigma ^2_A$, where ${\mathbf {A}}$ is the $n \times n$ pedigree-based numerator relationship matrix and $\sigma ^2_A$ is the additive genetic variance; and ${\mathbf {e}}$ is the residual vector whose elements are assumed to be independent and identically distributed as normal with zero mean and variance $\sigma ^2_e$. A commonly used technique for making predictions of yet-to-be-observed data is best linear unbiased prediction (BLUP) [39], where estimation of ${\mathbf {b}}$ and prediction of ${\mathbf {u}}$ are performed simultaneously. Variance components can be estimated, for example, by restricted maximum likelihood (REML).

If dense markers (e.g., SNPs) are available, the following model can be used for genome-enabled prediction (e.g., [41]):

2 $$\begin{aligned} {\mathbf {y}} = {\mathbf {1}}\mu + {\mathbf {Xb}} + \sum _{j=1}^p{\mathbf {w}}_j\alpha _j + {\mathbf {e}}. \end{aligned}$$

Here, *p* is the (possibly large) number of SNPs and the assumption is that the QTL that contribute to the phenotype ${\mathbf {y}}$ are in linkage disequlibrium (LD) with at least one SNP. In this model, $\alpha _j$ is the substitution effect of the $j{\mathrm{th}}$ SNP; ${\mathbf {w}}_j$ is an $n\times 1$ vector, whose elements $W_{ij}$ are the genotype code ($W_{ij}=0$, 1 or 2 for genotypes $A_2A_2$, $A_1A_2/A_2A_1$ or $A_1A_1$) of SNP *j* for the $i{\mathrm{th}}$ individual. One can also write $\big \{\sum _{j=1}^p{\mathbf {w}}_j\alpha _j\big \}$ as ${\mathbf {W}}\varvec{\alpha }$, where ${\mathbf {W}}$ is $n\times p$, whose $j{\mathrm{th}}$ column is ${\mathbf {w}}_j$, and $\varvec{\alpha }$ is $p\times 1$, whose $j{\mathrm{th}}$ element is $\alpha _j$. SNP effects can be learned in a Bayesian process (e.g., [41, 47]) by drawing samples from posterior distributions using Markov chain Monte Carlo (MCMC) techniques. Predictive performance using Model 2 is often better than for Model 1, and several studies have suggested that including both pedigree and marker information can achieve an even higher prediction accuracy [69, 70].

Models described above assume that QTL and SNPs are inherited in a Mendelian manner. However, in the presence of imprinting, or more generally, parent-of-origin effects, receiving one allele from the mother might have a different effect on ${\mathbf {y}}$ than receiving the same allele from the father [59, 60, 71]. Before the genomic era, the following mixed model using pedigree information was proposed to account for parent-of-origin effects [72, 73]:

3 $$\begin{aligned} {\mathbf {y}} = {\mathbf {1}}\mu + {\mathbf {Xb}} + {\mathbf {Zu}} + {\mathbf {Mg}} + {\mathbf {e}}, \end{aligned}$$

where ${\mathbf {y}}$, $\mu$, ${\mathbf {b}}$, ${\mathbf {u}}$ and ${\mathbf {e}}$ are as in Model 1; ${\mathbf {g}}$ is a $2n\times 1$ vector of additional genetic effects expressed only when inherited from a maternal or paternal gamete, assuming that ${\mathbf {g}}\sim N({\mathbf {0}}, {\mathbf {L}}\sigma ^2_g)$ with ${\mathbf {L}}$ being a $2n \times 2n$ gametic relationship matrix calculated from a known pedigree.

When considering SNPs, Shete and Amos [60] proposed the following one-locus model that regresses phenotype on the number of alleles received from a specific parent to account for parent-of-origin effects:

4 $$\begin{aligned} {\mathbf {y}} = {\mathbf {1}}\mu + {\mathbf {Xb}} + {\mathbf {I}}_{\venus }\alpha _{\venus } + {\mathbf {I}}_{\mars }\alpha _{\mars } + {\mathbf {e}}, \end{aligned}$$

where $\alpha _{\venus }$ and $\alpha _{\mars }$ are the average effects of receiving one $A_1$ allele from the female and male parents (maternal and paternal allele substitution effects), respectively, and ${\mathbf {I}}_{\venus }$ and ${\mathbf {I}}_{\mars }$ are vectors of associated indicator variables. Both $I_{i\venus }$ and $I_{i\mars }$ (the $i{\mathrm{th}}$ element of vectors ${\mathbf {I}}_{\venus }$ and ${\mathbf {I}}_{\mars }$, respectively) take values 0 or 1 so the combination of these two indicators gives the genotype codes of four genotypes. For example, $I_{i\venus }=I_{i\mars }=1$ indicates an $A_1A_1$ genotype and $I_{i\venus } = 1$, $I_{i\mars } = 0$ indicates an $A_2A_1$ genotype (maternally inherited allele is written first). This model can be extended to include all available SNPs simultaneously as in whole-genome prediction studies. Thus, we combined Models 3 and 4, which contain both pedigree and marker information, into a WGP model (called POE model hereafter) that is suitable for traits affected by parent-or-origin effects:

5 $$\begin{aligned} {\mathbf {y}} = {\mathbf {1}}\mu + {\mathbf {Xb}} + {\mathbf {Zu}} + {\mathbf {Mg}} + \sum _{j=1}^p{\mathbf {I}}_{j\venus }\alpha _{j\venus } + \sum _{j=1}^p{\mathbf {I}}_{j\mars }\alpha _{j\mars } + {\mathbf {e}}. \end{aligned}$$

To evaluate the performance of the POE model, it was compared with the additive model (referred to as ADD model hereafter) without parent-of-origin effects at either the pedigree or SNP levels. Model ADD is then:

6 $$\begin{aligned} {\mathbf {y}} = {\mathbf {1}}\mu + {\mathbf {Xb}} + {\mathbf {Zu}} + \sum _{j=1}^p{\mathbf {w}}_j\alpha _j + {\mathbf {e}}. \end{aligned}$$

## Data and model evaluation

### Mouse data

Some studies have suggested that obesity-related traits might be affected by imprinting in both humans [74] and mice [75]. An indicator of obesity, body mass index (BMI), was shown to be affected by parent-of-origin effects as well [76]. Hence, we chose BMI as the response variable in this study.

The data set used here is publicly available at http://mus.well.ox.ac.uk/mouse/HS/ and has been used in other studies (e.g., [69, 77]). It includes 1940 individuals that were obtained by crossing eight inbred strains, followed by 50 generations of approximately random mating. BMI measurements pre-corrected for body weight, season, month and day, and more than 12,000 genotyped SNPs located on 19 autosomes were collected. Additional description of this data set can be found from the data website and from [78]. In order to incorporate POE into the analysis, the two reciprocal heterozygotes $A_1A_2$ and $A_2A_1$ need to be distinguished from each other such that each allele of a SNP has a known parental origin. To do this, haplotype inference was performed using BEAGLE 3.3.2 [79, 80]. After this step, all SNPs with a minor allele frequency (MAF) less than 0.05 were removed, resulting in 10,021 SNPs for subsequent analyses.

Models POE and ADD (as in Eqs. 5 and 6 above, respectively) were used to perform whole-genome predictions of BMI. In this data, ${\mathbf {b}}$ included sex, litter size and cage density. Regarding the polygenic effect ${\mathbf {u}}$, Legarra et al. [77] and de los Campos et al. [69] conducted whole-genome prediction studies using the same mouse data and both suggested that including pedigree information in this data set provided no benefit in terms of predictive ability because the relationships among the full-sib families were relatively weak. Therefore, we dropped the polygenic term in the mouse data analysis. For the same reason, the term ${\mathbf {g}}$ was also dropped. Furthermore, a vector of random cage effects ${\mathbf {c}}$ with incidence matrix ${\mathbf {C}}$ was included in both models. ${\mathbf {c}}$ was assumed to be normally distributed with zero mean and variance–covariance matrix ${\mathbf {I}}\sigma ^2_c$.

### Simulated data

We used simulated data to evaluate the performance of the POE and of the ADD models under different situations. Parent-of-origin effects were simulated using the following two-step procedure. First, we used QMSim [81] to simulate a genome of 10 pairs of chromosomes each 1 Morgan long. Each chromosome had 1000 randomly located bi-allelic SNPs, so there were 10,000 SNPs in total, as in the mouse data. Approximately 150 simulated QTL were randomly located in the genome and these were not chosen from the simulated SNPs. QTL effects were randomly drawn from a normal distribution with zero mean and variance set to the software default value. The population started from 100 males and 100 females with 1000 generations of random mating to create LD between QTL and between SNPs and QTL; mutation rates were $u_{\mathrm {QTL}}=10^{-4}$ per QTL and $u_{\mathrm {SNP}}=10^{-2}$ per SNP, respectively. All QTL and SNP genotypes were fixed in generation 1. In the three most recent generations, without mutation, the population was expanded to 2000 individuals per generation with a 1:1 sex ratio.

In step 2, parent-of-origin effects were introduced by mimicking imprinting. For a long time, imprinting has been viewed as a “full-null” phenomenon, where the silencing of the imprinted allele is complete while the expression of the allele inherited from the other parent is intact; this is usually considered as the canonical definition of imprinting [71]. However, genomic imprinting can potentially operate at any level of gene regulation (e.g., at promoters, enhancers, splicing junctions, or polyadenylation sites, etc.) to present a more complex pattern of parent-specific differential expression [82]. For example, recent studies have provided evidence that, for some imprinted loci, both alleles are differentially expressed in a parent-of-origin-preferential or parent-of-origin-dependent manner [83], indicating that the silencing is incomplete [84, 85]. Such deviation from the canonical imprinting, defined as partial imprinting [30, 86], has been incorporated in the aforementioned one-locus imprinting model [17, 59, 60], and was also considered in our simulation. Let $\theta _{ij1}$ and $\theta _{ij2}$ (given by QMSim output) be the two allele effects of QTL *j* in individual *i* obtained from a certain QMSim run. Because QMSim records the parental origin of these two alleles, $\theta _{ij1}$ and $\theta _{ij2}$ can be represented by, say, $\theta _{ij\venus }$ and $\theta _{ij\mars }$, respectively. If this QTL is maternally imprinted, the genotypic value at this QTL for individual *i* can be written as:

7 $$\begin{aligned} \rho \cdot \theta _{ij\venus }+\theta _{ij\mars }, \end{aligned}$$

where $\rho$ is a parameter that controls the level of imprinting. Five different values were assigned to $\rho$: 0, 0.25, 0.5, 0.75, and 1, where $\rho =1$ corresponds to no imprinting, $\rho =0$ to complete imprinting, and $\rho =0.25, 0.5, 0.75$ define different levels of partial imprinting. We further assumed that a proportion $s=\{0.15$, 0.3, 0.45, 0.6} of $n_{\mathrm {QTL}}$ QTL were either paternally or maternally imprinted with equal frequency (a validation on the choice of these values is given in Discussion). Hence, the phenotypic value of individual *i* is:

8 $$\begin{aligned} y_i&= \sum _{j\in {\mathrm {NI}}}\big (\theta _{ij\venus } + \theta _{ij\mars }\big ) + \sum _{j\in {\mathrm {MI}}} \big (\rho \cdot \theta _{ij\venus } + \theta _{ij\mars }\big ) \\&\quad + \sum _{j\in {\mathrm {PI}}}\big (\theta _{ij\venus } + \rho \cdot \theta _{ij\mars }\big ) + \varepsilon _i, \end{aligned}$$

where NI, MI and PI are sets of $(1-s)\cdot n_{\mathrm {QTL}}$ non-imprinted, randomly selected $\frac{1}{2}s\cdot n_{\mathrm {QTL}}$ maternally imprinted and $\frac{1}{2}s\cdot n_{\mathrm {QTL}}$ paternally imprinted QTL, respectively, and $\varepsilon _i$ is the environmental effect on individual *i* given by QMSim. Note that the environmental effect $\varepsilon _i$ was not changed and that a common $\rho$ was shared by all imprinted QTL in a particular scenario for simplification.

Equation 8 was applied to the three recent generations (1001, 1002, and 1003) in all 20 combinations of $\rho$ and *s*. In subsequent analyses, generation 1002 was the training set and generation 1003 was the testing set. This whole procedure was replicated 5 times and the average predictive performance of all replicates was used for model evaluation.

### Model training and phenotype prediction

The additive relationship matrix ${\mathbf {A}}$ and the gametic relationship matrix ${\mathbf {L}}$ were calculated from the pedigree using the R package synbreed [87]. Both the ADD and POE models were trained with an implementation of MCMC using the R package BGLR [88, 89]. Each chain was run for 60,000 iterations, with the first 10,000 iterations discarded as burn-in and the rest were thinned by a factor of 10.

For the ADD model, the conditional prior distribution of the substitution effect of marker *j* was a normal distribution with zero mean and variance $\tau ^2_j\sigma _e^2$, where $\sigma _e^2$ came from a scaled inverted $\chi ^2$ distribution with scale $S_e$ and degrees of freedom $df_e$ set to default values in package BGLR [89]; $\tau ^2_j$ was drawn from an exponential distribution with parameter $\lambda ^2/2$. Hyperparameter $\lambda ^2$ was drawn from a Gamma distribution with shape *s* and rate *r* set to default values. This prior creates a double-exponential posterior density for marker effects, given $\lambda$, and is referred to as Bayesian Lasso [69, 90]. The infinitesimal additive effects ${\mathbf {u}}$ had a conditional multivariate normal prior $N({\mathbf {0}}, {\mathbf {A}}\sigma ^2_u)$, where $\sigma ^2_u$ was drawn from a scaled inverted $\chi ^2$ distribution with scale $S_u$ and degrees of freedom $df_u$ set to default values. Similarly, for cage effects, ${\mathbf {c}}|\sigma ^2_c\sim N({\mathbf {0}}, {\mathbf {I}}\sigma ^2_c)$ and again, the scale $S_c$ and degrees of freedom $df_c$ for the prior of $\sigma ^2_c$ were set to default values.

For the POE model, prior distributions were similar to those described above, except that two marker effects, the paternal and maternal allelic substitution effects, were included for each marker. The extra vector of gametic effects was assumed to have the distribution ${\mathbf {g}}|\sigma ^2_g,{\mathbf {L}},S_g,df_g\propto N({\mathbf {g}}|\sigma ^2_g,{\mathbf {L}})\cdot \chi ^{-2}(\sigma ^2_g|S_g,df_g)$. Again, all hyperparameters for the scaled inverted $\chi ^2$ distributions were set to package default values.

After model training, predictions were made on the testing set. Predictive correlation and predictive mean squared error (MSE) were the two metrics used for model evaluation.

## Results

### Mouse data analysis

The data set was randomly partitioned into training and testing sets according to the within-families approach of [77]. The cross-validation was repeated five times for stability assessment. Table 1 gives average results over the five replications. The ADD model performed slightly better than the POE model when evaluated by different metrics, but the difference was minimal. Our results with the ADD model were in agreement with those of [77] and [69], including the estimated variance components (Table 2).

**Table 1:** Average of testing set results of five cross validation replicates in the mouse data ( SE $=$ standard error)

Model	$Corr^{(P)}_{{\mathbf {y}},\hat{{\mathbf {y}}}} \, \hbox {(SE)}^{\mathrm{a}}$	$Corr^{(S)}_{{\mathbf {y}},\hat{{\mathbf {y}}}} \, \hbox {(SE)}^{{\mathrm{b}}}$	$\hbox {MSE (SE)}^{{\mathrm{c}}}$
ADD	0.321 (±0.067)	0.327 (±0.071)	0.00347 (±1.49$\times 10^{-4}$)
POE	0.309 (±0.059)	0.318 (±0.076)	0.00371 (±1.53$\times 10^{-4}$)

*ADD* additive model, *POE* parent-of-origin effects model

${}^{\mathrm{a}}$ $Corr^{(P)}_{{\mathbf {y}},\hat{{\mathbf {y}}}}$: Pearson’s correlation between observed and predicted value

${}^{\mathrm{b}}$ $Corr^{(S)}_{{\mathbf {y}},\hat{{\mathbf {y}}}}$: Spearman’s correlation between observed and predicted value

${}^{\mathrm{c}}$ $\hbox {MSE}$: Mean squared error

**Table 2:** Estimated variance components ($\times 10^{-4}$) in the two models with all individuals included

Model	$\hat{\sigma }^2_c$	$\hat{\sigma }^2_{e}$
ADD	3.37	17.89
POE	3.39	17.74

*ADD* additive model, *POE* parent-of-origin effects model

### Analysis of simulated data

In the simulation, five replicates were run, with each replicate resulting from an independent run of QMSim simulation. Each of the five realizations had training and testing sample sizes of 2000 individuals each; the number of SNPs was equal to 10,000 and the number of QTL in each replicate was equal to 142, 167, 158, 141 and 149, respectively.

**Table 3:** Comparison of predictive correlations (Pearson’s) among ADD, POE, and ADD-POE models

Data	$Corr^{(\mathrm {ADD{\text{- }}POE})}_{{\mathbf {y}},\hat{{\mathbf {y}}}} - Corr^{(\mathrm {ADD})}_{{\mathbf {y}},\hat{{\mathbf {y}}}}$	$Corr^{(\mathrm {ADD{\text{- }}POE})}_{{\mathbf {y}},\hat{{\mathbf {y}}}} - Corr^{(\mathrm {POE})}_{{\mathbf {y}},\hat{{\mathbf {y}}}}$
Mouse	0.000	0.005
$\hbox {Simulated}^{{\mathrm{a}}}$	0.001	−0.080

*ADD* additive model, *POE* parent-of-origin model, *ADD-POE* parent-of-origin model where two substitution effects are modeled only to markers with significant signals on parent-of-origin effects

${}^{{\mathrm{a}}}$ Takes $s=0.6$, $\rho = 0$ as a benchmarking scenario

**Fig. 1:**
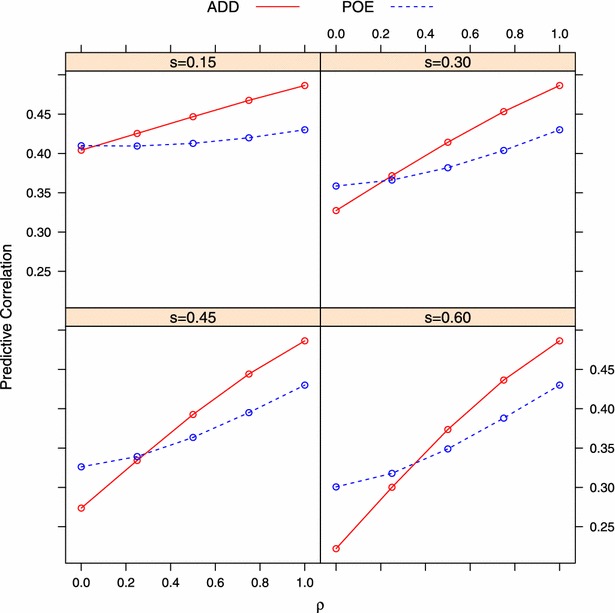
Average predictive correlation of two models measured by Pearson’s correlation $\big (Corr^{(P)}_{\hat{{\mathbf {y}}},{\mathbf {y}}}\big )$ between observed and predicted phenotype under different simulation settings. *ADD* additive model, *POE* parent-of-origin effects model. $s=$ proportion of imprinted QTL; $\rho =0$ and $\rho =1$ denote complete imprinting and no imprinting, respectively

**Fig. 2:**
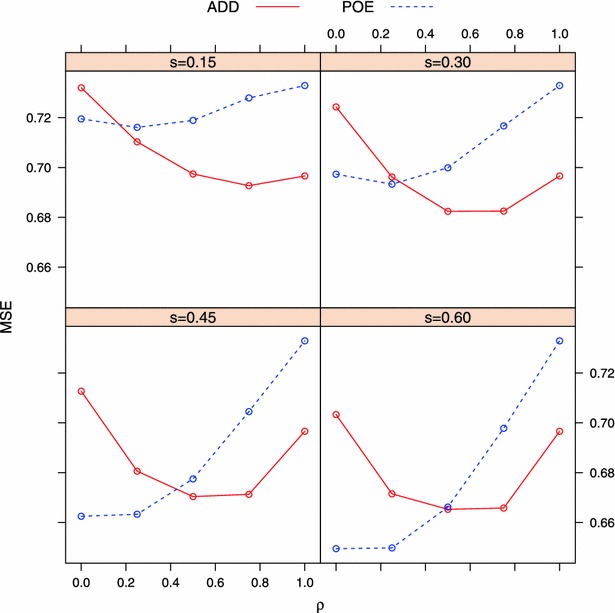
Averaged mean squared error (MSE) of two models between observed and predicted phenotype under different simulation settings. *ADD* additive model, *POE* parent-of-origin effects model. $s=$ proportion of imprinted QTL; $\rho =0$ and $\rho =1$ denote complete imprinting and no imprinting, respectively

As described in “Simulated data” section, each replicate had 20 scenarios, each corresponding to a combination of $\rho$ (imprinting level) and *s* (proportion of imprinted QTL). When $\rho =1$, however, three scenarios were redundant because in this case, all QTL were unimprinted such that different values of *s* made no difference (Eq. 8). Figure 1 displays the average prediction accuracy measured by Pearson’s correlation between observed and predicted phenotypes in different simulation scenarios, and Fig. 2 shows the MSE performance of the two models. Under both evaluation metrics, the ADD model performed better than the POE model when no imprinting was simulated ($\rho =1$). When there were no parent-of-origin effects, the *p* extra parameters in the POE model led to overfitting of the training data, thus sacrificing predictive ability of future data. With parent-of-origin effects, the POE model outperformed the ADD model but in a manner that depended on the *s* and $\rho$ settings. Typically, the POE model was better than the ADD model when $\rho$ was small and *s* was large.

## Discussion

Our results indicate that the POE model was not superior to the ADD model in terms of prediction when applied to the real mouse data. When using simulated data, however, our results showed that the POE model outperformed the ADD model under some circumstances, depending mainly on the choice of *s* and $\rho$. This result was consistent with a recent simulation study that incorporated imprinting effects in WGP [62]. Their prediction model was adapted from the one-locus imprinting model of [17, 59, 60]. However, instead of applying Bayesian regression directly by extending the one-locus imprinting model to include all available SNPs, the authors adopted a G-BLUP framework where genetic relationship matrices were generated for the additive, dominance, and imprinting effects. In their simulation, two parameters affected the performance of a prediction model with imprinting, namely the degree of imprinting and the number of imprinted QTL, which had the same role as $\rho$ and *s* in our simulation, and produced similar results as those obtained in our study. A discussion on our simulation results and related topics is provided in the following sections.

### Predictive performance of the ADD and POE models

#### Case 1: complete imprinting ($\rho =0$)

**Fig. 3:**
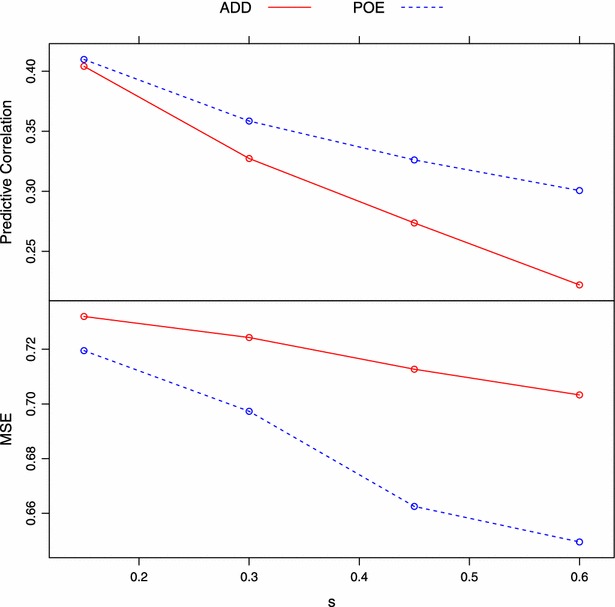
Trend of averaged predictive correlation and MSE with change of *s* (proportion of imprinted QTL) under $\rho =0$ (complete imprinting). Predictive correlation and MSE decrease as *s* goes up for both models. *ADD* additive model, *POE* parent-of-origin effects model

When imprinting was complete ($\rho =0$), it was not surprising that the POE model performed better than the additive ADD model. The superiority of the POE model over the ADD model depended on *s*, i.e., the larger the proportion of imprinted genes, the bigger the difference on predictive correlation and MSE between the two models. As *s* increased, a larger fraction of genetic variation was attributed to parent-of-origin effects, which cannot be captured by the ADD model. An interesting observation from Figs. 1 and 2 is that, for a given model, the predictive correlation and MSE decreased with an increase of *s* (Fig. 3). Recall that the data was simulated such that the allele effect was multiplied by $\rho$ (less imprinting as $\rho \rightarrow 1$), and fraction *s* of all QTL were assumed to be imprinted (Eq. 8). Suppose a QTL is maternally imprinted (the allele inherited from the mother written first), and that the values of the four genotypes (expressed as deviations from the population mean) are:

9 $$\begin{aligned} \begin{aligned} G_{11}&= \rho \cdot \theta _1 + \theta _1,\\ G_{21}&= \rho \cdot \theta _2 + \theta _1,\\ G_{12}&= \rho \cdot \theta _1 + \theta _2,\\ G_{22}&= \rho \cdot \theta _2 + \theta _2. \end{aligned} \end{aligned}$$

Let *p* and *q* be the frequencies of the $A_1$ and $A_2$ alleles. The genetic variance at this locus can be calculated as:

10 $$\begin{aligned} \sigma ^2&= P_{11}\cdot G^2_{11} + P_{21}\cdot G^2_{21} + P_{12}\cdot G^2_{12} + P_{22}\cdot G^2_{22} \\&=(1+\rho ^2)pq(\theta _1-\theta _2)^2, \end{aligned}$$

where $P_{ij}$ is the genotype frequency of $A_iA_j$ assuming Hardy-Weinberg equilibrium. Note that $\theta _1-\theta _2$ is $\alpha$, the allele substitution effect defined by a standard additive genetic model. From Eq. 10, when $\rho =1$ (no imprinting), the expression yields $2pq\alpha ^2$, the additive variance of a standard genetic model (e.g., [91, 92]). When $\rho < 1$, however, this variance (“signal”) decreases as $\rho$ approaches 0 (i.e., increased imprinting level). Hence, for a given value of $\rho$ that is smaller than 1 (0 in this case), the total variance of all QTL becomes smaller as *s* increases. Since the environmental distribution was the same in all settings, heritability decreased as *s* increased, producing a lower predictive ability.

#### Case 2: no imprinting ($\rho =1$)

**Fig. 4:**
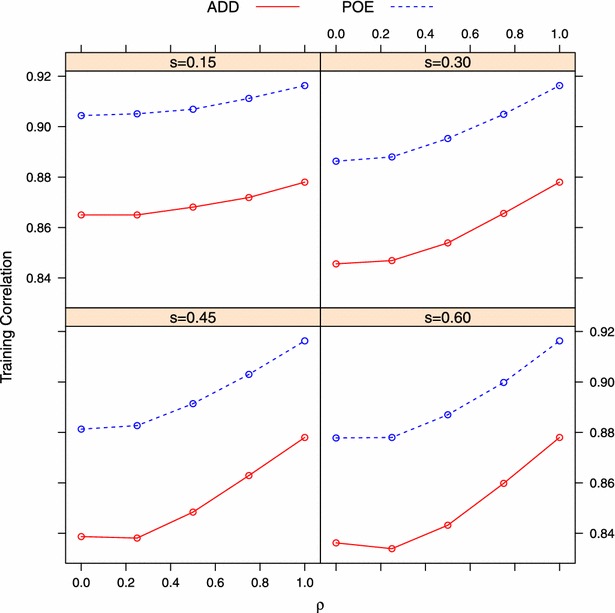
Training accuracy of two models measured by Pearson’s correlation $\big (Corr^{(P)}_{\hat{{\mathbf {y}}},{\mathbf {y}}}\big )$ between observed and fitted phenotype under different simulation settings. *ADD* additive model, *POE* parent-of-origin effects model. $s=$ proportion of imprinted QTL; $\rho =0$ and $\rho =1$ denote complete imprinting and no imprinting, respectively

As stated above, when $\rho =1$, the value of *s* does not affect the simulated data. In this simpler case, the ADD model outperformed the POE model in terms of predictive correlation and MSE, since the extra parameters in the POE model captured noise only. This is because, if, instead of capturing signal in the data, the better fit is due to higher model complexity, a penalty would be given to such a model during the testing process [93]. In our Bayesian implementation, genome-wide incorporation of parent-of-origin effects approximately doubled the number of parameters relative to the ADD model. This higher complexity provided a better fit to the data, as shown in Fig. 4: the training correlation of the POE model was always higher than that of the ADD model by about 4 %. However, a lower predictive correlation of the POE model ($\rho =1$, Fig. 1) indicated that the extra parameters in the POE model were not capturing model signal, at least when $\rho =1$. For the same reason, the POE model was expected to have a higher prediction error than the ADD model when no parent-of-origin effects affected the trait (Fig. 2).

Overfitting might be a reason why the ADD model was better as observed in the mouse data analysis and here in simulation when $\rho = 1$. Technically, a more complex model would enhance prediction if true underlying signals are captured by the extra parameters, so that overfitting is not an issue. However, if the true signal is not strong enough or training sample size is not large enough, overfitting would degrade prediction performance in the testing step. Although some parent-of-origin effects seem to exist in the mouse data, as indicated by the previous study using the same data [61], these are not strong enough to overwhelm overfitting, resulting in a lower predictive performance when the POE model was used.

#### Case 3: partial imprinting

**Fig. 5:**
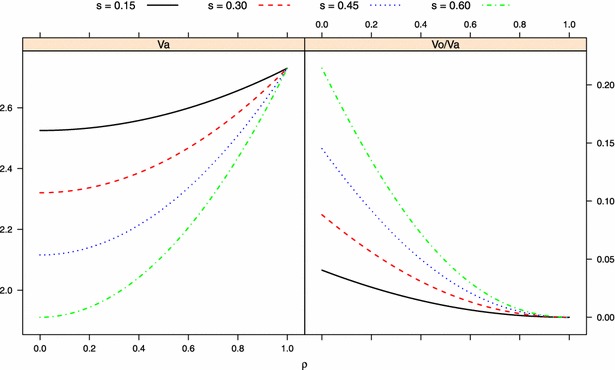
Stylized representation of the change of total additive variance across all 150 simulated QTL loci ($V_a$, *left panel*) and proportion of total additive variance due to parent-of-origin effects ($V_o/V_a$, *right panel*) at different values of $\rho$ (imprinting level, changes from 0 to 1) and *s* ($=\{0.15, 0.3, 0.45, 0.6\}$, proportion of imprinted QTL)

As imprinting changed from the highest ($\rho =0$, complete imprinting) to the lowest level ($\rho =1$, no imprinting), the predictive correlation of both models increased gradually for any value of *s*, since total additive variance (signal) increased during this course (Eq. 10; left panel of Fig. 5), so the predictive ability increased accordingly. Also, because the ADD model was better at $\rho =1$ but the POE model was better at $\rho =0$, curves representing the two models crossed at some point, and it was interesting to note that the value of $\rho$ associated with the cross point increased (representing a lower level of imprinting) as *s* went up (Fig. 1). Intuitively, the POE model would outperform the ADD model when the proportion of signal due to parent-of-origin effects reaches some threshold. Here, the variance accounted for by parent-of-origin effects is expressed as:

11 $$\begin{aligned} \sigma ^2_o = \frac{1}{2}pq(\theta _1-\theta _2)^2(1-\rho )^2 \end{aligned}$$

according to the four genotypic values in Eq. 9 and the one-locus imprinting model of [59, 60], and [17]; the ratio between Eqs. 11 and 10 gives the proportion of additive variance accounted for by parent-of-origin effect at that locus. For a larger *s*, this threshold is reached much faster than at a smaller *s* as $\rho \rightarrow 0$ (Fig. 5, right panel), indicating that when fewer QTL are imprinted, a higher level of imprinting is needed for the POE model to gain advantage, as expected.

### Overfitting and combining the ADD and POE models

Modeling all SNPs with two substitution effects each (i.e., $\alpha _{\venus }$ and $\alpha _{\mars }$) could be problematic since not all SNPs are subject to parent-of-origin effects and this could be the cause of overfitting, as observed previously. In order to circumvent the potential overfitting problem in modeling the parent-of-origin effects, it may be worth to detect SNPs that are strongly associated with parent-of-origin effects *a priori* and model two substitution effects for those SNPs only. Furthermore, as an extension of [72], we assumed that parental contributions from the paternal and maternal sides are independent with equal variance at the pedigree level. However, when there are no parent-of-origin effects, these two effects are likely to be correlated [94]; in this case the overparameterization at the pedigree level may reduce the predictive ability as well [62], and it may be worth to drop the gametic relationship term from the model. To implement this analysis, we first detected such parent-of-origin-effect-associated SNPs by using the method suggested in our previous study [61]. The following model (ADD-POE model) was then evaluated in the same cross-validation approach to assess if it improved the overfitting problem:

12 $$\begin{aligned} {\mathbf {y}}&= {\mathbf {1}}\mu + {\mathbf {Xb}} + {\mathbf {Zu}} + \sum _{j\notin \Phi }\mathbf {w }_j\alpha _j \\&\quad + \sum _{j\in \Phi }{\mathbf {I}}_{j\venus }\alpha _{j\venus } + \sum _{j\in \Phi }{\mathbf {I}}_{j\mars }\alpha _{j\mars } + {\mathbf {e}}, \end{aligned}$$

where $\Phi$ represents a set of markers with significant parent-of-origin effects at a 0.05 significance level after controlling for multiple testings using the Šidák’s correction.

Contrary to our expectation, Model 12 did not compromise prediction accuracy. Instead, the predictive performance of this model was only mildly better than the ADD model but much worse than the POE model in a simulation case where *s* is large and $\rho$ is small (Table 3). One possible reason for this result could be that although our simulation configured a relatively strong parent-of-origin case, the single-marker regression approach may still not be able to detect a large number of markers that are strongly associated with a true imprinted QTL (no replicates identified more than 50 significant SNPs), similar to all conventional GWAS studies. Therefore, the vast majority (i.e., ${>}99.5\,\%$) of all available SNPs will be modeled as additive instead of “imprinted”, and hence the improvement of the ADD-POE model over the ADD model was very limited.

The ADD-POE model attempted to find a possible source of overfitting and tried to handle it at both the pedigree and the SNP levels. Since these two factors could be confounded, we added the term ${\mathbf {Mg}}$ back to Model 12 and evaluated how the predictive ability changed. As a result, this model’s performance was almost identical to that of the ADD-POE model when using the simulated data, which indicated that when dense SNPs are used for prediction, modeling a relatively “rare” effect (here the parent-of-origin effects) across all SNPs may lead the model to suffer from severe overfitting, and overfitting due to this reason at the SNP level could be much larger than that due to an overparameterization at the pedigree level. Furthermore, our simulation chose QTL randomly and assigned a smaller “absolute” effect with a proportion parameter $\rho$ to reflect a non-equivalent contribution from the paternal and maternal genomes. Although this approach was able to introduce parent-of-origin effects, it may break some connection between the pedigree and the QTL that was established in the original QMSim simulation as well. This nearly identical predictive ability with or without the gametic relationship term as observed here could be the result of this disconnection. In order to better understand the behavior of gametic relationships, a more specific simulation approach that considers other mechanisms than imprinting would be helpful.

### Proportion of imprinted QTL

In our simulation, values of 0.15, 0.3, 0.45 and 0.6 were assigned to *s* (proportion of imprinted QTL) in different scenarios. These values were chosen arbitrarily and are much larger than the proportion of imprinted genes with available evidence since, among the approximately 25,000 human or murine genes, only about 200 have been identified as imprinted (http://igc.otago.ac.nz/home.html), i.e., 1 % of the total number of genes. Even the smallest value of *s* chosen (0.15) is too large compared to this small fraction observed to date.

However, this (i.e., about 200) is the number of experimentally identified imprinted genes, approximately. This means that the function, expression profile and regulating mechanisms of such genes were assessed in well-designed experiments, with verified imprinting status. It is possible that there are more imprinted genes in the mammalian genomes that have not been discovered so far. For example, Luedi et al. [95] and Brideau et al. [96] predicted that there might be hundreds of imprinted genes in the murine genome, although no consensus estimate on the number of imprinted genes in the mammalian genome is available [97]. Furthermore, imprinting might be more prevalent than previously assumed, as argued in several review studies (e.g., [13, 98]). Specifically, among 127 detected metabolic-related QTL, about 60 % had imprinting effects. In an earlier study, 54 % of 602 genes expressed in human kidney or liver tissues were shown to have strong parent-of-origin effects caused by preferential expression, with some of them not located in known imprinted genomic regions [99]. Therefore, based on these studies, we decided to increase the proportion of imprinted QTL in our simulation over the 1 % mentioned earlier.

Moreover, for the approximately 200 identified imprinted genes, a vast majority are growth- and/or development-related. This was shown when the famous “parent-offspring conflict hypothesis” was proposed [100–102] to explain the evolution of imprinting. Although Lush often stated the view that all complex traits are possibly affected by all genes at various degrees [103, 104], it is unlikely that all tens of thousands of genes in the mammalian genome affect a trait jointly [105]. Since there is no consensus on how many genes affect specific complex traits, tens to several hundreds might be a reasonable guess. Hence, within the hundreds of genes that control a single trait, say, fetal growth, it is possible that a considerable proportion is subject to imprinting. In addition, imprinting is a major cause of parent-of-origin effects, but not the only one [106]. Therefore, when imprinting was considered as the only cause to simplify the source of parent-of-origin effects in the simulation, we set the proportion of imprinted QTL up to 60 %.

### Other sources of information than DNA polymorphisms

Incorporating parent-of-origin effects into a prediction model may be helpful if it accounts for a considerable proportion of the total variance. In practice, additive variance is the major contributor to phenotypic variability for most complex traits [107]. Along with the overfitting problems associated with the POE model, the preceding implies that the POE model may bring only a minimal advantage in most cases. Therefore, it might be helpful to consider other sources of information in whole genome prediction to incorporate parent-of-origin effects. Since epigenetics, a main cause of parent-of-origin effects, is the study of heritable variation that does not involve a change of DNA sequence [108–110], our prediction model may fail under many situations because only variation at the DNA level (e.g., SNPs) is used as input. Hence, incorporating epigenetic information in addition to SNPs might be useful [111], as it has already been successfully used to identify disease-related genomic regions through epigenome-wide association studies (EWAS) [112–114].

Including epigenetic information in whole-genome prediction has been previously investigated and seems promising (e.g., [115, 116]). However, it can also be challenging. One aspect is the amount of information one needs to deal with. Consider DNA methylation as an example: it is the addition of a methyl group to either the 5-position carbon atom of the cytosine pyrimidine ring, or to the 6-position nitrogen atom of the adenine purine ring, with the latter observed mainly in mitochondrial DNA of flowering plants [117]. Two important features of DNA methylation are: (1) it is tissue and developmental-stage specific; (2) it is reversible, since the added methyl group can be removed from the methylated DNA. Due to this second feature, methylation status is unstable compared to DNA polymorphisms and, for a certain cytosine locus, it may shift between methylated and unmethylated states. Thus, although modern technologies are able to convert the unstable methylation information into stable sequence information via bisulfite treatment (e.g., [118, 119]), the methylation profile is for a specific time in a specific sample of cells. The term “methylome” is thus abused: in many studies, it actually refers to a “snap shot” of the entire methylome at a certain time point from a certain tissue given the first feature of DNA methylation. Compared to DNA sequence information which is size-invariant (unless a somatic mutation occurs) throughout an individual’s life time, the size of the methylome is highly variable and can be extremely large. Along with other epigenetic mechanisms such as histone modification, the size of the human epigenome is potentially enormous. For example, the diploid human epigenome contains more than $10^8$ cytosines (of which ${>}10^7$ are found in CpG dinucleotides, the major target of mammalian DNA methylation) and more than $10^8$ histone tails (the target of histone modification) that can all potentially vary [112]. It has been estimated that the human epigenome could be thousands of times larger than the genome [120]! Given this magnitude, choosing appropriate epigenetic information from a suitable tissue is crucial, and powerful and reliable analytical tools must be developed to ensure an appropriate use of the information.

Apart from the size of the epigenome, epigenetic mechanisms are affected by environmental effects. For instance, the methyl group added to a DNA molecule must come from a methyl group donor. One major source is the diet [121], so different diets can result in different methylation profiles that lead to different phenotypes. Several cases demonstrate the impact of nutrition on epigenetics. In mice, the coat color of genetically identical individuals showed variation when their mothers were fed with different diets during pregnancy [122, 123]. In honey bees, almost all female individuals in a colony are (almost, if not exactly) genetically identical. However, the royalactin found in the royal jelly turns one (and only one) individual into a queen and the others remain as workers [124]. In livestock, maternal diet during pregnancy can alter the DNA methylation of the fetus and, hence, result in changes in gene expression [125]. This evidence indicates that environmental variation brings extra difficulties to the already complicated epigenetic analysis.

Furthermore, epigenome profiling is very expensive. In the case of methylation, due to the massive number of CpG sites within the mammalian genome, high-resolution methylation profiles are very costly. Although reduced representation bisulfite sequencing (RRBS, [126]) can reduce the profiling costs by selecting a small proportion of representative CpG sites from certain regions (e.g., gene promoter regions) of the genome, methylation profiling of a large cohort (e.g., thousands of individuals in a WGP study) is still expensive, especially when multiple “snapshots” of the methylome need to be considered.

In short, epigenetic polymorphisms could contribute to genetic studies and open a door to a better understanding of biological systems. However, many challenges need to be resolved before this information can be efficiently used to advantage.

## Conclusions

We propose a model that is capable of incorporating parent-of-origin effects into whole-genome prediction using pedigree and DNA information. Our study on real and simulated data suggested that the POE model could be useful when parent-of-origin effects contributed a large proportion to the genetic variation, which was in agreement with a recent study that incorporated parent-of-origin effects in whole-genome prediction under a GBLUP framework [62]. In addition, our results draw attention to a possible overfitting problem when considering parent-of-origin effects in WGP with a Bayesian implementation, and indicate that one should be careful when using a POE model for prediction if the true signal attributed to parent-of-origin effects is weak in practice.

Owing to the discovery of more imprinted genes and of parent-of-origin-effects-affected complex traits, obtaining predictions that take parent-of-origin effects into account seems attractive. However, our simulation indicated that it did not always work well unless parent-of-origin effects contributed to the complex trait substantially. Hence, assessing the contribution of parent-of-origin effects to the total genetic variance (e.g., [127]) prior to model training might be helpful, as well as considering other sources of information than that from DNA polymorphisms (e.g., epigenetic variation) in evaluating parent-of-origin effects. Because many technical challenges need to be faced at the current stage of knowledge, future studies need to explore more effective prediction machines for parent-of-origin-effects-affected complex traits in animals, plants, and humans.
